# Regulated Arginine Metabolism in Immunopathogenesis of a Wide Range of Diseases: Is There a Way to Pass between Scylla and Charybdis?

**DOI:** 10.3390/cimb45040231

**Published:** 2023-04-18

**Authors:** Eleonora A. Starikova, Artem A. Rubinstein, Jennet T. Mammedova, Dmitry V. Isakov, Igor V. Kudryavtsev

**Affiliations:** 1 Laboratory of Cellular Immunology, Department of Immunology, Institute of Experimental Medicine, Akademika Pavlova 12, 197376 Saint Petersburg, Russia; 2 Medical Faculty, First Saint Petersburg State I. Pavlov Medical University, L’va Tolstogo St. 6–8, 197022 Saint Petersburg, Russia; 3 Laboratory of General Immunology, Department of Immunology, Institute of Experimental Medicine, Akademika Pavlova 12, 197376 Saint Petersburg, Russia; 4 School of Biomedicine, Far Eastern Federal University, FEFU Campus, 10 Ajax Bay, Russky Island, 690922 Vladivostok, Russia

**Keywords:** L-arginine, immune response, immunometabolism, COVID-19, infectious disease, oncology, autoimmunity, immunotherapy, enzyme-based therapy

## Abstract

More than a century has passed since arginine was discovered, but the metabolism of the amino acid never ceases to amaze researchers. Being a conditionally essential amino acid, arginine performs many important homeostatic functions in the body; it is involved in the regulation of the cardiovascular system and regeneration processes. In recent years, more and more facts have been accumulating that demonstrate a close relationship between arginine metabolic pathways and immune responses. This opens new opportunities for the development of original ways to treat diseases associated with suppressed or increased activity of the immune system. In this review, we analyze the literature describing the role of arginine metabolism in the immunopathogenesis of a wide range of diseases, and discuss arginine-dependent processes as a possible target for therapeutic approaches.

## 1. Introduction

In 1886, the German chemist Ernst Schulze and his assistant Ernst Steiger extracted arginine from yellow lupine germ buds for the first time. In 1899, Schulze and Winterstein synthesized arginine (L-Arg) from L-ornithine and cyanamide, and, in 1910, the L-Arg structure was confirmed by Sorensen [1]. In 1987, nearly 100 years later, arginine-derived nitric oxide (NO) was shown to be a factor regulating vascular tone. NO was classified as a physiologically active intermediate product of L-Arg to nitrite/nitrate conversion in macrophages and endothelial cells. The discovery of the fundamental role of NO-related compounds in the physiology of the human cardiovascular system resulted in the 1998 Nobel Prize being awarded to Robert F. Furchgott, Luis J. Ignarro and Ferid Murad [2].

L-Arg is a dibasic cationic amino acid involved in various metabolic pathways [3,4]. L-Arg is mainly derived from the three sources in the in vivo–dietary intake, endogenous de novo production from L-citrulline, or protein catabolism. Endogenous L-Arg is largely synthesized in the renal proximal tubules from L-citrulline generated from dietary L-Arg, in intestinal epithelium [5]. Plasma L-Arg concentrations range from 50 to 250 µM [4,6,7,8,9], which is much lower than in subcellular compartments, where its concentration level reaches 1 mM [10].

Evidence suggests L-Arg is an mTOR pathway-signaling molecule [11]. There are at least five arginine sensors (GPRC6A, SLC38A9, CASTOR1, CASTOR2, TM4SF5) reported in the literature [12,13,14,15]. mTOR is considered the key regulator of important cellular processes, including protein synthesis, proliferation, autophagy, lysosomal function, metabolism and inflammation. Research into the influence of L-Arg on mTOR regulation opens a new intriguing chapter in L-Arg investigation history [16].

In mammalian cells, at least eight transporters are involved in L-Arg trafficking across the plasma membrane [17]. L-Arg is metabolized by those four groups of enzymes: arginases, nitric oxide synthases (NOS), arginine decarboxylase (ADC) and arginine:glycinamidinotransferase (AGAT). Among them, arginases and NOS exist in several different isoforms.

Arginases are manganese-containing enzymes hydrolyzing L-Arg into L-ornithine and urea in the liver-urea cycle important for ammonia detoxification. L-ornithine is a substrate for ornithine decarboxylase (ODC) that initiates the synthesis of polyamines. Alternatively, ornithine aminotransferase (OAT) can metabolize L-ornithine into proline. These metabolites are involved in regeneration processes. Polyamines (putrescine, spermine and spermidine) are necessary for cell proliferation, whereas proline enriches collagen. It was established that arginase is expressed in many cell types, and its two isoforms catalyze the same biochemical reaction. Human arginase 1 (ARG1) is a cytosolic protein expressed mainly in hepatocytes, as well as in myeloid lineage cells. Human arginase 2 (ARG2), localized in mitochondria, widely expresses in extrahepatic tissues. Both enzymes share as low as 58% amino acid sequence homology, bearing a nearly identical structure within the catalytic site [18,19].

NOS metabolizes L-Arg to produce L-citrulline and NO. NOS isoforms differ in structure and mechanisms of activity regulation. Neuronal (nNOS) and endothelial NOS (eNOS) are constitutively expressed in non-immune cells: neurons, muscle, and endothelial cells. Their activity is regulated by Ca-dependent calmodulin binding as well as protein phosphorylation/dephosphorylation at the serine residue [20,21]. The enzymes usually produce NO in low concentration, which acts as an intracelullar signaling molecule [22] and regulates vascular homeostasis [23]. Inducible NOS (iNOS) expression has long been considered specific to immune cells, but recent studies show that the spectrum of the cells expressing iNOS is much broader [23] than earlier thought. Immune cells produce NO along with reactive oxygen species (ROS) to eliminate pathogens and tumor cells. NO and its derivatives (Reactive Nitrogen Species—RNS) act at micromolar concentrations nonspecifically on various targets, which may result in normal cell damage. Therefore, iNOS activity is highly regulated. Expression of iNOS induces in response to pro-inflammatory cues: bacterial toxins, as well as cytokines interleukin (IL)-1β, interferon γ (IFNγ), and tumor necrosis factor α (TNFα). On the contrary, IL-4 and IL-10, transforming growth factor β (TGFβ), downmodulate iNOS gene expression. Mechanisms regulating the bioavailability of intracellular L-Arg play an important role in regulating NOS-mediated NO synthesis [20,21].

ADC and AGAT are not involved in regulating immune cell functions. In mammals, ADC is highly expressed in brain tissues [24], whereas AGAT is found in the brain and heart [25,26,27]. ADC metabolizes L-Arg into agmatine, which is, in turn, converted by agmatinase into putrescine and urea [28]. Except for agmatine inhibitory effects on macrophage iNOS, little is known about the role of these enzymes in immune system [29].

L-Arg plays a crucial role in detoxification of ammonia—a protein breakdown product acts as a secretagogue and serves as a substrate for the synthesis of NO, an important signaling molecule that regulates vascular tone and cytotoxic functions of macrophages. L-Arg is also a precursor in the synthesis of L-ornithine and agmatine, creatine and polyamines. Metabolism of L-Arg is involved in immune cell regulation [30]. It is now clear that L-Arg metabolism is engaged in the pathogenesis of tumor growth, inflammation, infectious diseases, and fibrotic processes [31,32,33,34,35,36,37], as well as physiological immunodeficiencies in newborns and pregnant women [38]. In this review, we analyze literature data describing the role of L-Arg metabolism in immunopathogenesis in a wide range of diseases and potential therapeutic approaches aimed at regulating arginine-dependent processes.

## 2. L-Arginine Metabolism in Pathogenesis of Bronchial Asthma and Allergies

Allergic asthma is associated with increased airway reactivity and inflammation. Macrophages are the most abundant immune cells in the lungs (approximately 70% of immune cells) and play an important role in airway inflammation in asthma. It is suggested that high levels of T helper cell (Th) 2 type cytokines IL-4 and IL-13, typical of asthma, result in increases of type 2 inflammation and activate mast cells, basophils, eosinophils and M2 macrophages [39,40,41]. Macrophage polarization is largely associated with pathogenesis of allergic asthma. M1 macrophages are believed to be the major effector cell subset in non-allergic asthma. In addition, the M1 macrophage phenotype is associated with pathophysiology of severe asthma, especially in patients with a poor response to systemic corticosteroids. M2 macrophage type predominates in allergic asthma [42]. M2 macrophages bear a high level of ARG1 expression, and the enzyme is considered to be the main consumer of L-Arg in vivo, because the arginases-coupled reaction rate (Vmax) is 1000 times greater than the eNOS-coupled one [43]. Accordingly, Matysiak et al. [44] found that patients with bronchial asthma had decreased their L-Arg level in the peripheral blood, as confirmed additionally by Cottrill et al. in a study of children suffering from bronchial asthma [45]. Moreover, the level of L-Arg in children, prone to exacerbations, was lower than in those with controlled bronchial asthma. The cause is considered to be the increased expression of ARG1 found in tissue biopsies in lung epithelial cells [46]. Immunohistochemical studies showed that ARG1 expression was increased in lung mononuclear leukocytes [47]. Patients with bronchial asthma were observed to have an increased activity of peripheral blood ARG1 [48,49] and decreased NOS activity [49]. ARG1 hyperactivation may result in a deficiency of L-Arg used by NOS, so in the absence of the substrate, the enzyme dissociation leads to ROS generation [48]. Due to a shortage in NO production and increased ROS generation, lung tissue cells are exposed to oxidative stress associated with concomitantly elevated bronchial hyperreactivity [49]. Thus, clinical study data confirm the negative role of ARG1 in asthma. Diverse ARG1 genotypes are also linked to various levels of efficacy of beta-2-agonists used in bronchial asthma treatment [50].

Opposite data were obtained regarding mitochondrial ARG2 in the pathogenesis of bronchial asthma. The phenotype of severe asthma was shown to correlate with low ARG2 activity [51]. In addition, patients with a clinical picture of bronchial asthma were also shown to have a high ARG2 expression in the epithelial cells in biopsy specimens. ARG2 activity suppressed iNOS and airway inflammation. Moreover, a relationship between the immunoglobulin E (IgE) level and ARG2 expression in this pathology was also observed [51]. Patients with low ARG2 level were characterized by their peak IgE production [51], and immunoglobulin concentration positively correlated with the progression of respiratory symptoms [52]. The airway epithelial cells in patients with asthma showed a high mitochondrial content and adenosine triphosphate (ATP) level. Cells with higher ARG2 expression were found to bear a low level of phospho-STAT6, a downstream target in the IL-4 signaling cascade [53]. Also, it was shown that respiratory tract epithelial cells in patients with bronchial asthma had a high iNOS expression that was not detected in the mononuclear cells in bronchoalveolar lavage fluid. In addition, a high expression of argininosuccinate synthase (ASS1), responsible for de novo L-Arg synthesis, was found in the cytosol of airway epithelial cells [53].

The role of the mitochondrial arginase isoform ARG2 remains poorly understood. Presumably, it supports oxidative phosphorylation in mitochondria and is involved in IL-10-mediated anti-inflammatory activity. In macrophage studies, ARG2 was shown to be the IL-10 downstream-signaling mediator, inhibiting production of pro-inflammatory cytokines [54,55,56]. The activity of ARG2 downmodulates the hypoxia-induced factor 1α (HIF-1α) and IL-1β in vitro. HIF-1α and IL-1β were shown to be highly expressed in the lipopolysaccharide (LPS) -induced model of acute inflammation in ARG2 mice. ARG2 deletion brought about both higher HIF-1α and IL-13, eotaxin-1 and IL-5 levels [53]. ARG2 can downregulate the NO level, preventing uncontrolled cellular apoptosis triggered by the RNS [18]. IL-10-mediated induction of ARG2 is required to regulate mitochondrial dynamics and maintain macrophage oxidative phosphorylation by enhancing Complex II of electron transport chain activity. Thus, macrophage ARG2 exerted an anti-inflammatory effect in an autocrine manner [55]. Based on experimental data and clinical observations, it can be assumed that upregulated ARG2 suppresses airway inflammation, reduced Th2 response, HIF-1α expression, and improved cellular respiration.

A therapeutic approach aimed at regulating L-Arg metabolism in bronchial asthma provides controversial data. L-Arg supplementation was shown to be able to reduce the level of inflammatory mediators, such as TNFα [57], but it does not affect one of the key clinical parameters—frequency of asthma exacerbation [58]. Recently, it was shown that L-citrulline, as a precursor of L-Arg, supplemented by basic asthma therapy, [52] was beneficial. The patients who received L-citrulline showed better disease control and lung function. While ARG2 in bronchial asthma contributed to a milder disease course, whereas ARG1 exerted an opposite effect by aggravating symptoms, drugs blocking ARG1 can be considered as promising for downregulating inflammation and enhancing the efficacy of main therapy in bronchial asthma.

## 3. L-Arginine Metabolism in Cancer

**Figure 1:**
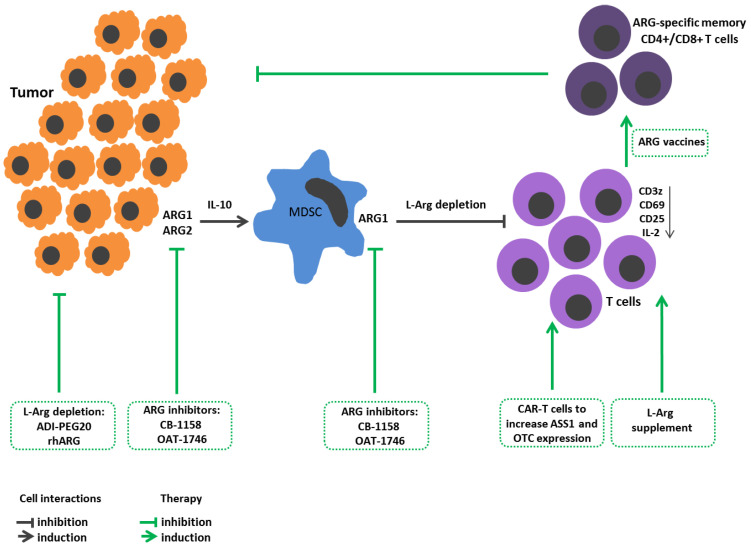
L-Arginine metabolism in tumor growth immunopathogenesis and arginine-dependent processes as a target of therapeutic approaches. Expression of arginases by tumor cells promotes their proliferation and metastasis. Arginase-mediated depletion of L-Arg in the tumor microenvironment contributes to the development of immunosuppression. Tumors also induce arginase in the microenvironment. Tumor-infiltrating arginase-expressing MDSC metabolites stimulate tumor mutagenesis, reinforce immunosuppression, decrease CD3z, CD25, CD69 expressions in T cells and IL-2 production. Therapeutic strategies: Arginine-hydrolyzing enzymes result in L-Arg exhaustion in tumor microenviroment and inhibit tumor cell proliferation. Administration of ARG inhibitors decreases tumor growth/metastasis. L-Arg supplement restores T cell functions. Reengineering CAR-T cells increases ASS1 and OTC expression to improve T cell L-arg bioavailability. Elaboration ARG1-specific CD4+ and CD8+ T cell promotes arginase-positive cell elimination. ADI-PEG20, pegylated arginine deiminase; ARG1, arginase 1; ARG2, arginase 2; ASS1, argininosuccinate synthase; CAR-T, chimeric antigen receptor T cells; L-arg, L-arginine; MDSCs, myeloid derived suppressor cells; OTC, ornithine transcarbamylase; rhARG, recombinant human arginase.

Being a proteinogenic amino acid, L-Arg is crucial for protein synthesis in actively proliferating cells, such as tumor cells [59,60,61] (Figure 1). L-Arg is also involved in other metabolic processes related to tumor growth, including production of NO, polyamines, nucleotides, proline, and glutamate. Moreover, it was shown that several enzymes of L-Arg metabolism (ODC) and amino acid transporters (CAT and SLC6A14) are actively involved in developing tumors [62].In contrast, it is known that immune cell activity also dramatically relies on L-Arg bioavailability [39,63]. Therefore, L-Arg metabolism has an ambiguous role in oncology.

Tumor cells require arginases for better proliferation and metastasis. For instance, ARG1 in neuroblastoma cells triggers expression of AKT and ERK signaling cascades leading to cell proliferation [64]. ARG2 in thyroid tumors elevates expression of proliferative markers, such as Ki-67, PCNA [65], whereas, in melanoma, it contributes to a promigratory cell phenotype with a high level of adhesion molecule ICAM-1 [66]. Patients with higher ARG1 expression showed less aggressiveness, invasiveness, metastasis, and higher differentiation of hepatocellular cancer cells [67]. Some studies report that high ARG2 expression in squamous cell carcinoma of the head and neck is associated with a poorer outcome [68].

At the same time, L-Arg deprivation during tumor growth becomes one of the mechanisms for reprogramming immune responses with induced immunosuppression. Therefore, arginase-expressing tumor cells can simultaneously solve two issues, on the one hand, generating metabolites necessary for growth, and, on the other hand, reprogramming the tumor microenvironment to suppress the immune reactions.

Some tumors implement these strategies by inducing arginase in their microenvironment [69,70]. That was supported in the study by Lian et al. showing that colorectal cancer cells co-cultured with monocytic myeloid leukemia cells (THP-1) with upregulated ARG1 expression in the latter, and that colorectal cancer cells contributed to monocyte differentiation towards the anti-inflammatory M2 cells secreting IL-10 [71]. Tumor-infiltrating arginase-expressing monocyte lineage cells can induce tumor immunoresistance [70]. A high expression of ARG1 is found in peripheral blood myelocytes of patients with breast cancer [72].

In the late 1990s, myeloid lineage cells in the tumor microenvironment were described. They differed from macrophages, had immature neutrophil and monocyte morphology, and exhibited a strong suppressive activity against T cells. These cells were dubbed myeloid-derived suppressor cells (MDSCs). It is now recognized that MDSCs comprise two main well-defined subsets: mononuclear (M-MDSC) and polymorphonuclear (PMN-MDSC) MDSCs. Paulo C. Rodriguez et al. characterized such cells as mature myeloid cells with high arginase activity [73]. Murine studies demonstrated that inhibition of arginase with Nor-NOHA exerted antitumor effects in vivo. MDSCs, along with arginase, possess multiple mechanisms to regulate immune cell function, including production of NO, peroxynitrite, superoxide and hydroxyl peroxide, prostaglandin E2, TGFβ, and adenosine [74]. The accumulation of MDSCs is associated with a negative clinical outcome in cancer patients, as well as a poor response to various immunotherapeutic strategies [74]. Low plasma L-Arg levels were linked to increased surface expression of the inhibitory costimulatory molecule PD-L1 on myeloid lineage leukocytes and worse cancer survival [75]. It is suggested that MDSCs migrating to the lymph nodes may restrain T cell activation and clonal expansion and thereby terminate immune response during infection [76]. MDSCs are also considered an essential player in regulating immune response in chronic inflammation, trauma and autoimmune diseases [74].

L-Arg is necessary for lymphocyte activation and proliferation, as well as execution of related effector functions during immune response. Availability of L-Arg ex vivo within a physiological range modulates CD3z expression levels [39,77,78], using a mechanism not yet fully elucidated [4]. The regulation of CD3z expression is a crucial mechanism for modulating T cell activation. L-Arg deprivation affects TCR signaling by reducing F-actin level and disrupting the immune synapse structure in activated T cells [79]. L-Arg depletion also prevents nuclear translocation of NF-κB (nuclear factor κB) p65 [80]. L-Arg deficiency lowers IL-2 production and the expression of early activation markers CD25 and CD69 in cultured human T cells [81]. A sufficient amount of L-Arg is a necessary condition for T lymphocyte entry into the cell cycle [82]. L-Arg demand markedly elevates upon T cell activation, and the amino acid supplement promotes CD4+ and CD8+ T cell survival and antitumor activity [83]. It was shown that, in the absence of L-Arg, NK cell granule exocytosis and cytotoxicity are completely abrogated, and cytokine secretion and proliferation are profoundly suppressed [84]. A high expression of arginases in a tumor microenvironment suppresses T cell response. A tumor microenvironment deficient in L-Arg contributes to developing cytopenia because this amino acid is required for proliferation of myeloid and CD34+ hematopoietic cells [70].

Two opposing strategies are used in tumor therapy targeting L-Arg metabolism. One strategy is to increase L-Arg bioavailability to immune cells for stimulating an antitumor immune response [85]. L-Arg added at supraphysiological concentration stimulates antitumor activity of CD8+ T cells not only in a cell culture, but also in vivo upon reinfusion into tumor-bearing mice [83]. Remarkably, T cells encountering L-Arg deficiency deploy mechanisms to maintain the intracellular amino acid reserves [86] by upregulating ASS1 for resynthesis of L-Arg from L-citrulline [87]. Therefore, reengineering chimeric antigen receptor T cells (CAR-T) to increase ASS1 and ornithine transcarbamylase (OTC) expression promotes persistence of CAR-T cells in vivo and their activity against solid and hematological tumors [88]. Hence, CAR-T therapy combined with enhanced L-Arg bioavailability can increase antitumor therapeutic efficacy. Introducing L-Arg supplementation may also potentiate M1 macrophage antitumor activity, because L-Arg is the sole substrate in the iNOS-mediated NO generation [89].

Recently, new drugs—arginase inhibitors—have been tested. Arginase inhibitor-substance CB-1158 has performed well in experiments on mice and cell cultures. The mechanism of the drug action is aimed at blocking ARG1 in MDSCs, resulting in T lymphocyte active proliferation and increased CD8+ T cell tumor infiltration [90]. Another drug, OAT-1746, a selective inhibitor of ARG1/ARG2, had no effect on viability but suppressed the growth of tumor cells, abrogated tumor metastasis, and enhanced the anti-PD1 antibody-related antitumor effect in mouse models and in vitro [91].

ARG1-specific IFNγ-producing Th1 CD4+ T cells were found in the mononuclear fraction of peripheral blood in patients with melanoma [92]. CD8+ T cells from melanoma patients also showed an immune response against ARG1. These ARG1-specific CD4+ and CD8+ T cells predominantly displayed the memory cell phenotype (CD45R0+) [93]. Based on these data, the generation of so-called “arginase vaccines” inducing arginase-specific T cell clones is considered among the promising strategies in antitumor therapy. When ARG1 peptide vaccine was tested in mice, the data indicated enhanced immune response against tumor cells. It was also shown that, combined with PD-1 inhibitors, such vaccination induces a more potent antitumor response [94]. In 2022, a human trial with ARG1 peptide vaccine demonstrated its safety. At the same time, nine (90%) out of ten patients had measurable peptide-specific reactions in the peripheral blood, and in two (20%) out of ten patients, the disease was stabilized during treatment [95].

Another therapeutic approach for oncological diseases is aimed at suppressing tumor cell proliferation, metastasis, and invasion and, paradoxically, proposing L-Arg deprivation. The most common current method of amino acid deprivation is supposed to use arginine-hydrolyzing enzymes. The best studied drugs are presented by pegylated arginine deiminase (ADI-PEG20) and recombinant human arginase (rhArg).

ADI-PEG20, via L-Arg depletion, was shown to induce autophagy and subsequent cancer cell death [96]. ADI-PEG20 worked well in stage II clinical trials in patients with hepatocellular carcinoma [97]. Also, ADI-PEG20 was highly effective in ASS1 negative melanoma patients [98]. The best effect was observed while using ADI-PEG20 with chloroquine [99] or cytostatics, as they inhibit autophagy, and more tumor cells undergo apoptosis. Thus, when using ADI-PEG20 with cisplatin, a more intense apoptosis was observed in melanoma cells [100]. The use of ADI-PEG20 has its drawbacks and limitations. Since L-citrulline is one of the metabolites of ADI, ASS1/ASL-positive tumors are resistant to therapy with this drug [101,102], while some ASS1-negative tumors acquire the ability to express ASS1 after ADI-PEG20 treatment [99,103].

Arginases, unlike ADIs, metabolize L-Arg into ornithine and uric acid, rhArg, which affects ASS1-positive tumors [101]. A pegylated counterpart (Peg-rhARG1) showed positive results in a phase I clinical trial in patients with hepatocellular carcinoma [104].Furthermore, rhArg coupled to an IgG1 Fc fragment (rhArg-Fc) is currently being assessed in vitro [105]. Autophagy inhibitors, including chloroquine and bafilomycin A1, potentiate rhArg- and ADI-PEG20-induced cytotoxicity [105].

## 4. L-Arginine Metabolism in Autoimmune Diseases

In autoimmune diseases, both genetic and environmental factors contribute to impaired tolerance to self-antigens [106], resulting in the formation of autoreactive B and T cells and tissue damage.

**Figure 2:**
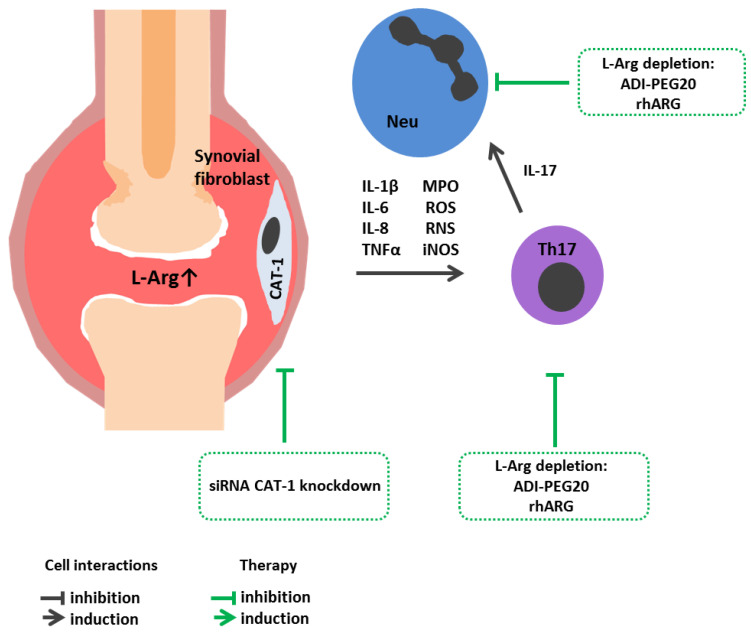
L-Arginine metabolism in rheumatoid arthritis immunopathogenesis and arginine-dependent processes as a target of therapeutic approaches. Increased synovial fluid L-Arg in RA patients correlates with IL-1β, IL-6 and IL-8 levels. A high level of CAT-1 in sinovial fibroblasts increases L-Arg bioavailibility. A high L-Arg level promotes Th17 polarization and activation, which supports neutrophilic inflammation and cartilage destruction. Therapeutic strategies: CAT-1 knockdown with siRNA inhibite L-Arg uptake. L-Arg exhaustion with arginine-hydrolazing enzymes affects Th17 clonal expansion, iNOS activity and RNS production. ADI-PEG20, pegylated arginine deiminase; CAT-1, cationic amino acid transporter-1; IL, interleukin; L-Arg, L-arginine; Neu, neutrophil; rhARG, recombinant human arginase; siRNA, small interfering RNA; Th17, T helper 17.

Rheumatoid arthritis (RA) is a chronic inflammatory autoimmune disorder that primarily affects joints. In RA, immune cell joint infiltration, synovial hyperplasia as well as excessive secretion of pro-inflammatory cytokines, leads to cartilage degradation and bone erosion [107]. Lu et al. [108] revealed an increased L-Arg in the synovial fluid of RA patients. A positive correlation between L-Arg level and increased pro-inflammatory cytokine IL-1β, IL-6 and IL-8 level was observed (Figure 2). A further study showed that fibroblast-like synoviocytes in RA vs. osteoarthritis patients highly expressed CAT-1 (cationic amino acid transporter-1), the main L-Arg transporter. Moreover, CAT-1 knockdown with siRNA or inhibited L-Arg uptake with D-arginine markedly suppressed L-Arg metabolism, cell proliferation, migration, and secretion of cytokines in in vitro cultured fibroblast-like synoviocytes in RA [108]. In addition, increased activity and level of expression of ARG1 observed in the peripheral blood of patients with RA were inversely related to the Th17 cell level, playing a leading role in the pathogenesis of this diease [109]. Other studies showed that RA patients vs. control subjects had a significantly lower plasma level and L-Arg bioavailability [110]. Similar results were reported by Chandrasekharan et al., showing reduced levels of L-Arg and L-citrulline along with increased arginase activity in RA patients as compared to control group [111]. It is possible that L-Arg level in peripheral blood and/or synovial fluid in RA is associated with amino acid consumption by immune cells, which depends on the disease stage and severity. It may be expected that limiting the intake of exogenous L-Arg would reduce the inflammatory autoimmune response, as well as the fibroblast-like synoviocyte proliferation in RA patients.

Systemic lupus erythematosus (SLE) is a chronic autoimmune disease characterized by the production of autoantibodies against nuclear antigens, immune complex deposition, and tissue damage found in the kidneys, skin, heart, and lungs [112]. In SLE, there was a decreased level of peripheral blood L-Arg, which is supposed to be associated with an elevated level of NO metabolism leading to oxidative stress in SLE patients [113]. Recently described in patients with SLE, MDSCs with increased ARG1 expression could be responsible for Th17 differentiation, at least in vitro, and inhibition of ARG1 contributed to alleviated disease activity [114].

Crohn’s disease is a chronic granulomatous inflammatory disorder able to affect any part of the gastrointestinal tract, predominantly the terminal ileum and adjacent colon, and is characterized by a segmental, asymmetric distribution of granulomatous inflammation. The main clinical symptoms are presented by abdominal pain, diarrhea, fistulas, anal lesions, and systemic symptoms of varying severity [115]. L-Arg and ADMA were reduced in patients with active Crohn’s disease compared to control subjects and patients with active ulcerative colitis [116]. A decreased L-Arg level directly in inflamed tissues during ulcerative colitis could be associated with high *NOS2* activity, decreased ARG1 functions and expression [117].

In autoimmune pathologies, it is generally reasonable to use therapy aimed at L-Arg depletion to suppress immune cell activation. However, in terms of the underlying pathogenesis, autoimmune diseases represent a rather heterogeneous group. For example, in systemic scleroderma, L-Arg may be used as a substrate for NO synthesis in Raynaud’s syndrome to enable vasodilation to improve microcirculation [118]. At the same time, treatment aimed at either arginine depletion or L-Arg supplementation should be used with caution in any disease where endothelial dysfunction is involved [21].

## 5. L-Arginine in Neurodegenerative Diseases

Multiple sclerosis (MS) is a chronic inflammatory and neurodegenerative autoimmune disease that affects the central nervous system. Multiple sclerosis is characterized by immune dysregulation resulting in CNS infiltration with immune cells causing demyelination [119]. Furthermore, there is an increase in NOS activity and free NO level both in serum and cerebrospinal fluid samples [120]. On the other hand, arginase expression and activity are profoundly reduced, not only in patients with clinically isolated syndrome, but also in patients with relapsing-remitting multiple sclerosis [121,122]. Some studies showed a decline blood serum L-Arg level in MS patients [123,124]. The revealed decrease in the amino acid level is associated with its role as a precursor of an NO neurotransmitter, of which the production is markedly increased during lesion-inflammatory processes [125]. The decrease in L-Arg level may also result from elevated ARG1 activity reported in MS patients [126]. In other studies, no significant differences in the concentration of blood serum and cerebrospinal fluid free L-Arg was found in patients with various forms of MS and healthy controls [127,128].

The pathogenesis of Alzheimer’s disease [129] is characterized by a developing neurotoxic inflammatory process [130]. Publications on the role of L-Arg metabolism in Alzheimer’s have demonstrated, for instance, that ARG1 mRNA level was increased in the cortex of the frontal lobe in patients with Alzheimer’s disease, without affecting ARG2 level [131]. However, another study obtained diametrically opposite results indicating that in the frontal cortex of patients, the level of ARG2 mRNA increased whereas ARG1 expression level remained unchanged [132]. In murine models, arginase inhibition was observed to prevent cognitive decline in Alzheimer’s disease [133].

Recent studies on murine models of Alzheimer’s disease have shown a link between ARG1 deficiency in microglial cells and amyloid formation [37,134,135,136]. For example, Hunt et al. showed that overexpression of ARG1 in the CNS inhibited Tau-associated kinases (pGSK-3, CDK5p35, p38MAPK), contributing to Tau-protein phosphorylation and beta-amyloid deposition [134]. Furthermore, ARG1 induced autophagy of cortex and hippocampus cells by inhibiting mTOR [134]. Other studies have found that ARG1+ microglia cells phagocytized beta-amyloid components and contributed to amyloid plaque reduction [135]. Ma et al. proved that the repression of ARG1 in microglia cells inhibited phagocytosis of beta-amyloid and thereby increased amyloid plaques deposition that goes in line with the data above [136]. The authors suggest that low levels of ARG1 contributed to the activation of the mTOR-pathway due to L-arginine accumulation [136]. In other studies, it was shown that the arginase inhibitor prevented Tau-protein phosphorylation and cognitive decline in Alzheimer’s disease [133].

## 6. L-Arginine Metabolism in Infectious Diseases

### 6.1. Sepsis

The systemic immune response to infection is often accompanied not only by profound imbalance in the activity of arginine-metabolizing enzymes, but also by impaired endogenous L-Arg synthesis and exogenous dietary intake [137]. Patients with sepsis have a reduced level L-Arg in blood plasma, whereas potentiated protein catabolism is a key source of amino acids [137]. Patients at an intensive care unit (ICU) recovered after sepsis were found to have lowered de novo L-Arg synthesis. Moreover, in such critical conditions, both arginase and iNOS often become activated with increased production of urea and L-ornithine, as well as NO and L-citrulline [138]. A decrease in L-Arg level in plasma and cerebrospinal fluid was observed in patients with neonatal sepsis and meningoencephalitis [139]. Due to an increased arginase activation and a deficiency of L-Arg, the immune response to infection in septicemia deteriorates. It was recently shown that a high level of ARG1 in septic patient neutrophils suppresses the CD8+ T cell response by reducing the proportion of IFNγ- and granzyme B-expressing CD8+ T cells. The inhibition of ARG1 in vitro resulted in increased granzyme B + IFN-γ + CD8 + T cell and TNFα + IFNγ + CD8 + T cell levels [140]. It was further uncovered that sepsis patients had an increased number of MDSCs of varying phenotypes (CD14 + HLA-DR- monocytic (M)-MDSC, CD14-CD15+ low-density granulocytes/granulocytic (G)-MDSCs). G-MDSCs had a high level of ARG1 expression, which contribute to suppressed T cell response in sepsis [141].

Conversely, L-Arg supplementation maintains immune homeostasis, especially for T cell and macrophage functions. Patients receiving an arginine-enriched diet were shown to restore T cell functioning that had been impaired due to trauma or surgery during cardiovascular diseases [142,143]. An L-Arg-rich diet in patients with sepsis at the ICU showed alleviated bacteremia and mortality [144].

Although NO is beneficial and critical to normal biological functions, its overproduction in endotoxic shock causes vasodilation and hypotension, resulting in subsequent death [145,146]. Suppressed NO production by L-Arg-catabolizing enzymes is considered a new approach in sepsis treatment [147]. ADI is currently being considered as a tool for suppressing the growth of ASS1\ASL negative tumors [148]. At the same time, a few timid attempts have been taken to use this enzyme in pathologies associated with hyperactivation of the immune system. For example, the use of ADI in sepsis has been tested in some studies [149]. An important feature of ADI is that one of the metabolites of this enzyme is the L-Arg precursor—L-citrulline [150]. This leaves room for ASS1\ASL-positive cells (e.g., endothelial cells) to resynthesize L-Arg from L-citrulline. It is believed that eNOS is the main consumer of L-citrulline-derived L-Arg [151]. Preliminary studies show that ADI inhibits the activity of iNOS, producing NO in high concentrations [152,153], but enhances the activity of eNOS, which generates the metabolite in homeostatic concentrations [154]. Theoretically, the ADI can act as a biochemical shunt, redirecting L-Arg from arginases and iNOS to eNOS. If this suggestion is correct, ADI can be used to treat conditions with NO overproduction and/or arginase hyperactivity without harming the homeostatic, vasoprotective functions of eNOS. This hypothesis is confirmed by recent studies which showed that ADI-PEG20 resulted in the depletion of L-Arg with the production of isomolar amounts of L-citrulline. This L-citrulline has the potential to be utilized by the L-citrulline recycling pathway, regenerating L-Arg and sustaining the amino acid availability in surrounding tissue [155]. It also seems promising to use the enzyme in allergic and autoimmune diseases. However, no studies have been conducted so far.

It is believed that in protracted critical conditions, such as sepsis, the use of L-Arg supplementation is beneficial due to its anabolic and antimicrobial effects [145]. However, therapeutic approaches aimed at regulating L-Arg metabolism in infectious diseases have their own characteristics. On the one hand, L-Arg deficiency due to suppressed immune responses may contribute to a longer circulation of infectious agents in the blood-stream, resulting in toxic shock. On the other hand, an L-Arg supplement may stimulate the immune system and provoke the development of vascular collapse due to increased NO production. This is supported by the data that L-Arg did not improve local perfusion and organ function [146] and could reduce blood pressure via an NO vasodilatory effect [147]. A decision about applying such supplements should be made individually depending on the patient’s condition. An amino acid supplement is not recommended at early stages of sepsis and in septic shock [127].

### 6.2. Viral Infections

**Figure 3:**
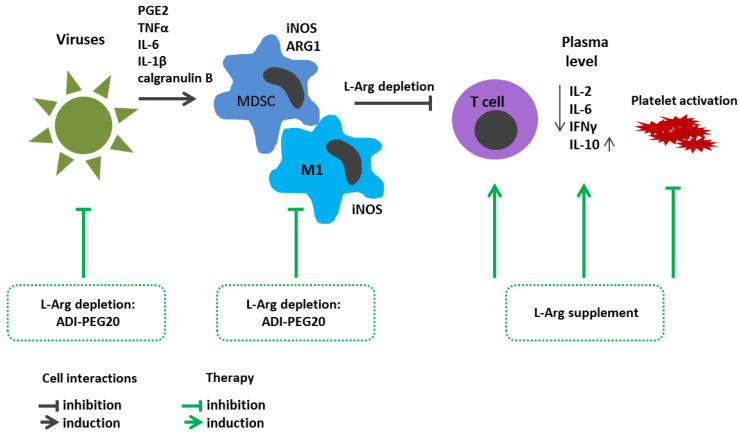
L-Arginine metabolism in viral infection immunopathogenesis and arginine-dependent processes as a target of therapeutic approaches. Inflammatory cues such as prostaglandin E2, TNFα, IL-6, IL-1β and calgranulin B contribute to MDSC expansion. MDSCs, expressing high levels of ARG1 and iNOS, limit L-Arg availability for proliferating T cells. Decreased blood L-Arg level results in platelet activation. L-Arg supplement reduces peripheral blood level of IL-2, IL-6, and IFNγ and increases IL-10. Therapeutic strategies: L-Arg deprivation inhibits viral replication. ADI-PEG20-mediated L-Arg depletion suppresses NO production and level of inflammatory response. ADI-PEG20, pegylated arginine deiminase; ARG1, arginase 1; IFNγ—interferon γ; IL, interleukin; iNOS, inducible nitric oxide synthase; L-Arg, L-arginine; M1, M1 macrophage; MDSCs, myeloid derived suppressor cells; PGE2, prostaglandin E2; TNFα, tumor necrosis factor α.

Previously, it was shown that in patients with hepatocellular carcinoma and a hepatitis C virus infection, a forced ADI-PEG20-mediated plasma L-arg depletion suppressed NO production and level of inflammatory responses [156] (Figure 3). The amino acid supplement was shown to stimulate the replication of some viruses and enhance cell infection, including that of members of Herpesviridae and Adenoviridae in vivo and in vitro [157,158]. Apparently, the positive effect of L-Arg depletion in viral infections is due to the dependence of biosynthesis processes on the amino acid bioavailability. Indeed, under conditions of L-Arg deficiency, proliferation of different cell types is suppressed precisely due to GCN-2 kinases activation along with suppressed global protein synthesis, which also affects the synthesis of viral proteins [156]. Therefore, L-Arg deprivation in patients with acute COVID-19 has been considered for some time as a promising approach that could retard SARS-CoV-2 replication in host cells [157,159].

Upon infection with SARS-CoV-2 virus, profound alterations were observed in the metabolism of individual amino acids including L-Arg [160]. Thus, it was shown that the level of blood serum L-Arg in patients with severe COVID-19 was significantly lower than that of patients with mild COVID-19 and the control group [161,162,163]. Moreover, a decline in L-Arg level was characteristic not only for adult patients, but also for children withSARS-CoV-2 infection [164,165].

One possible cause for the decreased L-Arg levels in COVID-19 patients could be MDSC accumulation. The main factors contributing to MDSC expansion in pathological settings are considered inflammatory cues such as prostaglandin E2, TNFα, IL-6 and IL-1β, as well as calgranulin B (S100A9). By producing ARG1 in combination with high levels of iNOS, MDSCs limit L-Arg availability to proliferating T cells. MDSCs delay viral clearance by inhibiting T cell proliferation and response, using various mechanisms, such as anti-inflammatory cytokine secretion, the suppression of IFNγ production or L-Arg depletion [166]. On the one hand, MDSCs, by reducing activation of immune responses against pathogens, restrain and mitigate potential collateral damage to the host organism. On the other hand, owing to ability to suppress effector T cells, MDSCs can interfere with the development of an immune response, thereby contributing to pathogen persistence and chronic infection [167]. While MDSC-related immunosuppressive properties may help restore tissue homeostasis and prevent hyperinflammation in an infection, the cells appear to have a pathogenic effect in severe COVID-19 [166]. Reizine et al. found an inverse relationship between the level of circulating CD3+ T cells and blood serum L-Arg in COVID-19 patients [161]. Within the total pool of peripheral blood leukocytes, patients with COVID-19 showed a decreased T cell count along with disease progression, whereas an increased proportion of such cells could be considered as a favorable sign [168,169]. The patient’s T cell level in the circulation was reduced both in comparison with control group and COVID-19 convalescent patients [170,171,172]. A dynamic T cell reduction as well as CD3+ CD4+ and CD3+ CD8+ cells was characteristic of patients with severe vs. moderate disease course [173,174], and the subset profile of both CD4+ and CD8+ T-lymphocytes underwent dramatic changes [175,176]. The detected alterations in T cell differentiation and “polarization” could, among others, be associated with alteration in relevant proliferative activity [177]; however, at least in vitro, supplementation with additional L-Arg restored CD3+ cell proliferative potential [161].

Another important consequence of decreased blood L-Arg level in patients with acute COVID-19 is platelet activation [178], which is reflected in upregulated surface PAC-1 expression that peaks typically in severe COVID-19. L-Arg deficiency also leads to increased platelet adhesion and decreased NO production, resulting in vasoconstriction and hypercoagulability [179].

Oral administration of exogenous L-Arg vs. placebo in routine therapy in severe COVID-19 markedly reduced hospital stay and ventilatory support [180]. Moreover, it was recently shown that exogenous L-Arg substantially reduced peripheral blood level of pro-inflammatory cytokines IL-2, IL-6 and IFNγ and increased concentration of anti-inflammatory IL-10 in patients with COVID-19 [181]. The data obtained on the change in balance of pro- and anti-inflammatory cytokines were confirmed by clinical observations showing that along with L-Arg supplement, the discharge time from the hospital was shortened and the need for respiratory support was also decreased [181]. In contrast, another study showed that applying L-Arg for patients with severe COVID-19 pneumonia had no marked effect on disease outcome [182]. Currently, special attention is also paid to long-term disorders—long or post COVID-19 syndrome—in the functioning of various organs and tissues in acute COVID-19 convalescent patients, not only in severe [183], but also in mild or even asymptomatic disease [184]. It should also be noted that a combination of L-Arg and vitamin C vs. a placebo applied for 28 days reduced patient fatigue and endothelial dysfunction associated with post-COVID-19 [185]. Positive effects of L-Arg supplementation in COVID-19 convalescent subjects were found as well [186].

T cell dysfunction in COVID-19 can be a result of decreased blood L-Arg level. This was indirectly confirmed by studies on immune status in patients with an active hepatitis B infection [187]. Peripheral blood CD8+ T cells of patients with active chronic hepatitis B reduced proliferative activity in response to TCR stimulation. Despite this, relevant T lymphocytes actively secreted TNFα and IFNγ and expressed cytotoxicity markers such as CD107a. Presumably, these effector functions were exerted by CD27-CD45RA + CD8+ T cells. Moreover, it was shown that intrahepatic CD8+ T lymphocytes produced less IL-2 in patients with chronic hepatitis vs. control subjects [187] who might not be compensated even by CD4+ T cells. Patient liver biopsies showed increased arginase activity and decreased plasma L-Arg than for the control. Intrahepatic CD8+ T lymphocytes selectively downregulate CD3ζ chain expression. When the cells were cultured with L-Arg in vitro, CD3ζ chain expression was restored and the proliferative potential of CD8+ T cells was increased, indicating that L-arg deficiency affected antiviral immune response at the site of inflammation [187].

Analyzing the level of L-Arg in severe fever with thrombocytopenia syndrome (SFTS) associated with bunyavirus infection (SFTSV) also reveals a decrease in blood serum L-Arg level [188]. L-Arg deficiency was associated with decreased intraplatelet NO level, platelet activation, and thrombocytopenia. Moreover, such patients had an increased level of G-MDSCs, expressing arginase at a high level. It was hypothesized that the functional activity of MDSCs was closely associated with suppressed activity of diverse CD3+ T cell subsets, suggesting an important role of arginase activity and L-Arg deficiency in impaired viral clearance.

### 6.3. Bacterial Infections

Tuberculosis brought about by *Mycobacterium tuberculosis* (MTB) is the leading cause of death worldwide [189]. However, progress in preventing TB epidemics is still slow, and it is doubtful that TB infections will be eradicated at this pace in the coming decades. Patients with tuberculosis were found to have highly expressed enzymes responsible for L-Arg catabolism in granulomas [190,191]. In *M. tuberculosis* infection, there was a decrease in peripheral blood L-Arg level [192]. Some studies showed that M1 macrophages expressing iNOS produce a high level of NO, especially effective against mycobacterial infection. A study with transgenic mice deficient in *Nos2* showed increased vulnerability to MTB infection [193]. In addition, macrophages that respond to MTB infection do not fit into the “M1” and “M2” binary macrophage classification. MTB infection uncovered macrophages co-expressing iNOS and ARG1 [191,194,195]. Although, iNOS has a higher affinity for L-Arg, ARG1 is featured with a higher maximum substrate utilization rate [9]. Thus, macrophages expressing ARG1 probably play an inhibitory role by limiting NO production and MTB clearance. ARG1-expressing macrophages were found in granulomas from MTB-infected patients and non-human primates [190,191]. Interestingly, loss of ARG1 in *Nos2−/−* mouse hematopoietic cells resulted in increased necrotic granulomas and increased mycobacterial load [196]. A mouse model lacking ARG1 in hematopoietic cells showed increased macrophage NO production and increased mycobacterial clearance [194]. In addition, the presence of ARG1 in granulomas correlated with reduced T cell proliferation [196]. Thus, it is likely that ARG1 acts to reduce lung T cell-mediated inflammation in MTB infection. Arginase-expressing G-MDSCs were shown to massively infiltrate the lungs upon infection with hypervirulent mycobacteria, promoting bacterial growth and development of inflammatory and necrotic lesions [197].

That the synthesis of L-Arg by immune cells is vital for control of mycobacterial load has been confirmed in experiments aimed at blocking its endogenous synthesis. Chimeric bone marrow mice with hematopoietic cells from ASS1 hypomorphic animals were unable to control MTB infection compared to controls [195]. In addition, mice with conditional hematopoietic or myeloid-targeted deletion of ASL or ASS1 showed an increased bacterial load after a challenge with Mb BCG or MTB [198]. The lack of L-Arg reduced the efficiency of the antigen presentation, as well as B cell activity and differentiation [189]. Using experimental mouse models, it was demonstrated that, in mycobacterial infections, L-Arg plays an important role in developing T cell immune response because it is necessary for T cell clonal expansion and differentiation, wherein antigen-presenting cells serve as the major source of L-Arg in the lymph nodes [199]. On the other hand, L-Arg deficiency leads to developing oxidative stress and DNA damage, which contributes to MTB death [200,201].

According to the results of clinical trials, orally administered L-Arg in patients with MTB-infection vs. placebo was accompanied by weight gain, higher sputum conversion and faster decline in symptoms such as cough [202]. On the other hand, an arginine-enriched diet in TB patients resulted in no prominent clinical changes [203]. The data obtained indicate that the method of L-Arg delivery to patients also markedly impacts its bioavailability. Moreover, similar results were obtained in another placebo-controlled study when daily L-Arg supplementation to the diet of TB patients had no effect on the clinical course of the disease [204]. An alternative approach to control mycobacterial infection could be based on blocking L-Arg pathways, which should also be used in clinical practice [205].

Non-tuberculous mycobacterial pulmonary diseases (NTM-PDs) are the most common clinical manifestations of NTM infections, often caused by a slow growing *Mycobacterium avium* complex (MAC) and a fast-growing *Mycobacterium abscessus* complex, including *M. abscessus* subsp. *abscessus (M. abscessus)*, *M. abscessus* subsp. *massiliense (M. massiliense)* and *M. abscessus* subsp. *bolletii (M. bollettii)*. Studies show that NTM patients demonstrate an impaired potential of Th1/Th17 differentiation and dysregulated IFNγ/IL-12B axis, a crucial immune pathway associated with NTM infection. It was found that the L-Arg level was reduced in the sera of patients with NTM-PD and mice infected with NTM. The oral administration of L-Arg profoundly reduced the NTM bacterial load in vivo and enhanced IFNγ-producing T cell responses, as well as iNOS expression in mice. The comparison of serum metabolomes from NTM-PD patients and control subjects revealed a markedly lower level of L-Arg, but not L-citrulline, in patients infected with *M. abscessus* or *M. massiliense*. In contrast, serum urea levels were substantially higher in NTM-PD patients than in controls, suggesting a shift towards M2 macrophages in the former. Similarly, *M. abscessus*-infected mice showed elevated serum urea levels after 3 weeks of infection. Pre- and post-infection oral treatment of NTM-infected mice with L-Arg markedly reduced the bacterial load and pathological inflammation in lung tissues. L-Arg supplement triggered host-protective immune responses by reshaping intestinal microbiota by enrichment with *B. pseudolongum*. Thus, the administration of L-Arg in NTM infection was beneficial and resulted in an increased activity of M1 macrophages, probably due to IFNγ-producing T cells [206].

A decline in the circulating L-Arg level was observed in infections of children and adults with malarial plasmodium [207,208] and could be associated both with increased L-Arg consumption by parasite arginase and increased activity of host arginases [209,210]. It is believed that increased plasma arginase activity in children with severe malaria is primarily due to the activated mononuclear phagocytes expressing this enzyme [211]. It was found that the absolute count of circulating G-MDSCs was prominently increased in severe malaria than in the control group. G-MDSC levels in uncomplicated vs. severe malaria were comparable [212]. Moreover, low L-Arg level was strongly associated with high parasitemia and severe malaria. Another study showed that hypoargininaemia in malaria infections also correlates with reduced NO production and *P. falciparum*-triggered erythrocyte deformability in vitro [213]. In addition, L-arg supplementation in malaria contributed to the recovery of microvascular functions [214].

## 7. Pathogen Immunoevasion Strategy via Altered Host L-Arginine Metabolism

**Figure 4:**
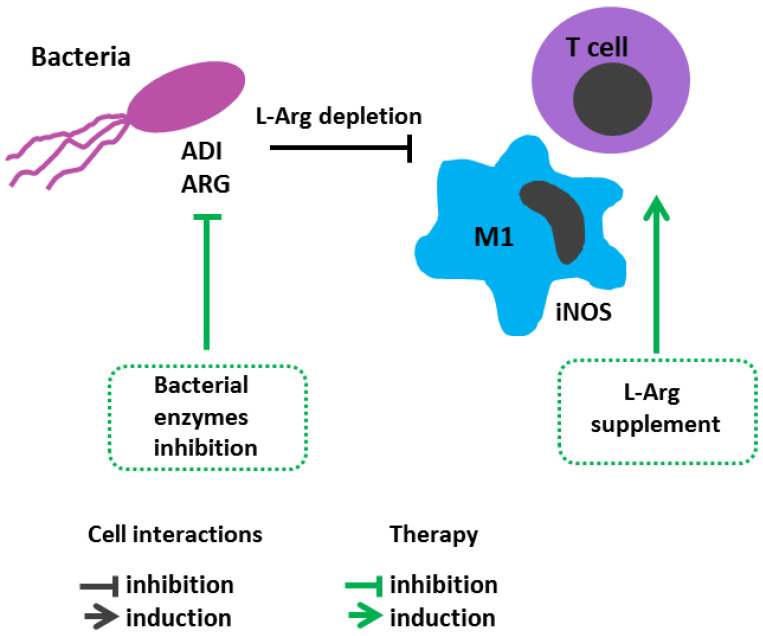
L-Arginine metabolism in bacterial infection immunopathogenesis and arginine-dependent processes as a target of therapeutic approaches. Pathogenic microbes affect L-Arg metabolism to reprogram host immune responses. Reduced L-Arg level suppresses T cell response, results in substrate depletion for iNOS, thereby reducing NO production.Therapeutic strategies: Oral administration of L-Arg reduces bacterial load and enhances T cell responses, as well as iNOS activity.ADI-PEG20, pegylated arginine deiminase; ARG, bacterial arginase; iNOS, inducible nitric oxide synthase; L-Arg, L-arginine; M1, M1 macrophage.

The key L-Arg role in regulation of immunity is underlined by evidence that pathogenic microbes also affect its metabolism to reprogram host immune responses [215,216] (Figure 4). If a pathogen drives a host L-Arg turnover via an arginase pathway, the process results in substrate depletion for iNOS, thereby reducing NO production. *Salmonella typhimurium*, [217] *Mycobacterium tuberculosis*, [194] *Leishmania mexicana* [218] and *Schistosoma mansoni* [219] deploy this strategy to survive in the host. *Salmonella* enhances ARG2 expression in macrophages. The inhibition of arginase using NOHA in mouse salmonella infections increased substrate availability for iNOS, reduced the bacterial load, and curbed infection in an NO-dependent manner [217]. A reduced MBT load was observed in ARG1-deficient mice, also showing that liver granulomas in BCG-infected mice produced more bactericidal nitrotyrosine after host arginase inhibition [194]. Interestingly, some pathogens, such as *Helicobacter pylori*, express *rocF* genes encoding arginase and depleting L-Arg as well as NO production by synthesizing their own enzyme [220]. *H. pylori* arginase can also directly impair T cell function by downregulating CD3z expression during infection [80]. Arginase is also responsible for collagen synthesis and tissue regeneration [221]. It is believed that cancer-causing pathogens such as *H. pylori* and the hepatitis C virus (HCV) may use arginase to modulate the host cell cycle from resulting in a cancerous condition. Data confirm that anti-arginase siRNA inhibits HCV ability to stimulate hepatocellular growth [222]. Elevated levels of salivary arginase were also reported in periodontal diseases, which decreased after antibiotic treatment [223]. To avoid macrophage attack after ingestion, *Candida* spp. applies the strategy of inducing its own intracellular arginase and urea amidolyase, able to contribute to pathogen survival by reducing nitrosative stress via iNOS inhibition [224].

ADI, another microbial arginine-hydrolyzing enzyme, utilizes L-Arg for pathogen survival at low pH at infection site or inside phagolysosomes being involved in ATP synthesis under low glucose conditions [225]. The PEGylated recombinant protein modification is being actively investigated as a drug for antitumor therapy [226,227]. Relatively few works are aimed at assessing an effect of this enzyme on protective immune responses. A study by Zachary T. Cusumano et al. demonstrated a new virulence mechanism in *S. pyogenes* found to use ADI to suppress NO production by host macrophages via L-Arg depletion. In vitro studies with supernatants of sonicated *S. pyogenes* M49-16 and its isogenic mutant (*S. pyogenes* M49-16delarcA) with inactivated ADI gene *arcA* confirmed the results reported by Cusumano et al. [152,153]. Mouse model of subcutaneous streptococcal infection showed that a decrease in plasma L-arg concentration was accompanied by thymus involution, increased thymocyte apoptosis and Treg cell differentiation [228]. *S. pyogenes* M49-16delarcA displayed reduced virulence compared to the original *S. pyogenes* M49-16 strain, revealing an important role of this enzyme as a streptococcal pathogenicity cue [228]. Streptococcal ADI was found to affect leukocyte inflammatory infiltrate in a murine air pouch model [229]. The ability of streptococcal ADI to suppress human peripheral blood lymphocyte proliferation in vitro was verified [230]. It should be noted that this enzyme is expressed by many, including highly pathogenic microbes, such as *Pseudomonas aeruginosa* [231], *Listeria monocytogenes* [232]. Further ADI investigation may contribute to better understanding of fine mechanisms underlying an interplay between pathogen and host immune system during diverse infections.

Whereas a competition between host and pathogen for L-Arg can determine an outcome of infectious diseases, then a therapeutic intervention aimed at regulating arginine-metabolizing enzymes may turn out to be a useful tool and a good alternative to antibiotics in numerous infections.

## 8. Injuries and Surgical Interventions

**Figure 5:**
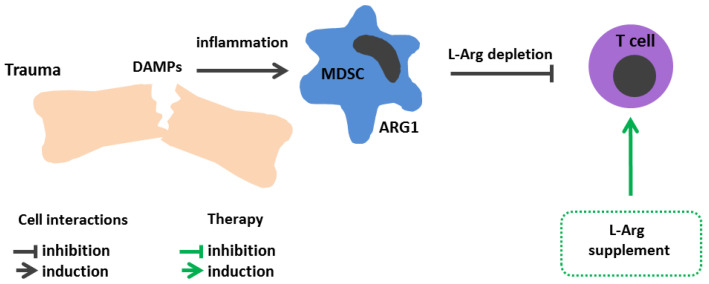
L-Arginine metabolism in trauma immunopathogenesis and arginine-dependent processes as a target of therapeutic approaches. MDSC differentiation is associated with excessive accumulation of DAMPs and other inflammatory mediators. The MDSC number, building up in injuries, accounts for decline in blood L-Arg and subsequent immunosuppression. Therapeutic strategies: L-Arg supplement increases lymphoproliferative response, wound healing and lowers the risk of infectious complications. ARG, arginase; DAMPs, danger associated molecular patterns; iNOS, inducible nitric oxide synthase; L-Arg, L-arginine; MDSCs, myeloid derived suppressor cells.

An elevated MDSC number, documented in a range of acute inflammatory conditions, including traumatic brain injury, blunt chest trauma, and burns, is considered to be one of the main reasons for decline in peripheral blood L-Arg level in these conditions [233,234,235,236] (Figure 5). Although the role of MDSCs has been well characterized in cancer, the clinical significance of this leukocyte population in injuries and sepsis remains unclear. The classical hypothesis regarding a mechanism of MDSC induction is based on the “two-signal model” described by Gabrilovich et al. [237]. According to this model, the expansion of immature myeloid cells is triggered by various growth- and colony-stimulating factors (granulocyte colony-stimulating factor, granulocyte-macrophage colony-stimulating factor, macrophage colony-stimulating factor), which activate the transcription factors C/EBPβ, STAT (signal transducer and activator of transcription)—3, and Notch suppressing terminal cell differentiation. The release of the second group of pro-inflammatory stimuli (high-mobility group protein B1, PGE2, NF-κB, STAT1, STAT6) and ER-stress signals stimulates MDSC expansion [238].

Although the proposed model on MDSC differentiation remains debated, it is hard to deny that the overproduction of such cells is associated with the excessive accumulation of inflammatory mediators and the immune system attempting to correct the imbalance. It is assumed that MDSCs are destined to suppress excessive inflammation provoked by abundantly released danger-associated molecular patterns (DAMPs). However, MDSC accumulation may “tip the scales in the opposite direction” and contribute to immune suppression, as well as an elevated risk of nosocomial infections [238].

Mader et al. analyzed the level of L-Arg and its metabolizing enzymes in peripheral blood and cerebrospinal fluid in patients with cerebral hemorrhages [239]. It turned out that, in addition to decreased L-Arg, there was an early increase in the NOS level followed by its decline to the baseline, whereas arginase activity was elevated throughout the follow-up period [239]. These data are consistent with previous studies in a cohort of ICU trauma patients [240]. These patients had elevated peripheral blood mononuclear cell ARG1 activity throughout their ICU stay. Furthermore, patients with injuries were shown to have a reduced peripheral blood L-Arg level. Likewise, the level of L-Arg in the peripheral blood also tended to decrease in burns [240]. L-arg-containing supplements are often used in ICUs for various injuries [138]. For instance, L-Arg used on burn patients increased their lymphoproliferative response, accelerated wound healing, markedly lowered C-reactive protein level, and contributed to a shorter ICU stay [240]. An L-Arg supplement is also used in preoperative patients’ diets, resulting in a lowered risk of infectious complications and a shortened hospital stay [241].

## 9. Conclusions

In recent years, it has been increasingly evident that, apart from a well-described role in various cardiovascular diseases, L-Arg also acts as an inflammatory modulator in various pathologies. Targeting L-Arg metabolic pathways can help regulate immunological responses. The use of L-Arg hydrolyzing enzymes, as well as relevant inhibitors, has limitations because these enzymes perform a number of homeostatic functions. eNOS is engaged in NO production, crucial for vascular biology. Arginase, being a key enzyme in the urea cycle, is involved in ammonia detoxification and regulates T cell functions. While there is an increasing L-Arg bioavailability to stimulate immune cell defense mechanisms, it inevitably helps the foe (tumor or pathogen) on the other side of the barricades. Reduction of the amino acid bioavailability compromises the effectiveness of immune responses. Again, while there is an increasing L-Arg bioavailability for NOS, the arginase activity invariably increases. L-Arg supplementation or depletion may negatively affect the vascular endothelium, especially in endothelial dysfunction with impaired eNOS activity. To effectively impact pathological events, a deeper insight into the L-Arg metabolic pathways in each specific pathology is required. Thus, development of a therapy aimed at regulating L-Arg metabolism remains a difficult task, serving as a path between Scylla and Charybdis.

## Data Availability

Not applicable.

## Abbreviations

ADC: arginine decarboxylase; ADI, arginine deiminase; ADI-PEG20, pegylated arginine deiminase; ADMA, asymmetric dimethyl arginine; AGAT, arginine:glycinamidinotransferase; ARG1, arginase 1; ARG2, arginase 2; ASL, argininosuccinate lyase; ASS1, argininosuccinate synthase; ATP, adenosine triphosphate; Baf A1, bafilomycin A1, CAR-T, chimeric antigen receptor T cells; CAT, cationic amino acid transporter; CQ, chloroquine; eNOS, endothelial nitric oxide synthase; DAMPs, danger associated molecular patterns; HCV, hepatitis C virus; HIF-1α, hypoxia-induced factor 1α; ICAM-1, intercellular adhesion molecule-1; ICU, intensive care unit; IFNγ—interferon γ; IL, interleukin; iNOS, inducible nitric oxide synthase; L-Arg, L-arginine; MDSC, myeloid-derived suppressor cell; MS, multiple sclerosis; MTB, *Mycobacterium tuberculosis*; NF-κB, nuclear factor κB; NK, natural killer; nNOS, neuronal nitric oxide synthase; NO, nitric oxide; NOHA, N-ω-nitro-L-arginine; NOS, nitric oxide synthase; NTM-PDs, non-tuberculous mycobacterial pulmonary diseases; OAT, ornithine aminotransferase; ODC, ornithine decarboxylase; PCNA, proliferating cell nuclear antigen; PD-L1, programmed cell death ligand 1; PsA, psoriatic arthritis; RA, rheumatoid arthritis; rhArg, recombinant human arginase; RNS, reactive nitrogen species; ROS, reactive oxygen species; SFTS, severe fever with thrombocytopenia syndrome; siRNA, small interfering RNA; SLE, systemic lupus erythematosus; STAT, signal transducer and activator of transcription; TBI, traumatic brain injury; TCR, T cell receptor; TGFβ, transforming growth factor β; Th, T helper cell; TNFα, tumor necrosis factor α.
